# Loading of Au/Ag Bimetallic Nanoparticles within and Outside of the Flexible SiO_2_ Electrospun Nanofibers as Highly Sensitive, Stable, Repeatable Substrates for Versatile and Trace SERS Detection

**DOI:** 10.3390/polym12123008

**Published:** 2020-12-16

**Authors:** Menghui Wan, Haodong Zhao, Lichao Peng, Xueyan Zou, Yanbao Zhao, Lei Sun

**Affiliations:** Engineering Research Center for Nanomaterials, Henan University, Kaifeng 475004, China; wanmenghui950825@163.com (M.W.); zhd880880@163.com (H.Z.); zouxueyan@henu.edu.cn (X.Z.); zhaoyb902@henu.edu.cn (Y.Z.)

**Keywords:** Ag/Au nanoparticles, synergetic enhancement, electrospun nanofibers, SERS substrates, versatile detection

## Abstract

In this paper, we propose a facile and cost-effective electrospinning technique to fabricate surface-enhanced Raman scattering (SERS) substrates, which is appropriate for multiple analytes detection. First of all, HAuCl_4_∙3H_2_O was added into the TEOS/PVP precursor solution, and flexible SiO_2_ nanofibers incorporated with gold nanoparticles (SiO_2_@Au) were prepared by electrospinning and calcination. Subsequently, the nanofibrous membranes were immersed in the tannic acid and 3-aminopropyltriethoxysilane solution for surface modification through Michael addition reaction. Finally, the composite nanofibers (Ag@T-A@SiO_2_@Au) were obtained by the in-situ growth of Ag nanoparticles on the surfaces of nanofibers with tannic acid as a reducing agent. Due to the synergistic enhancement of Au and Ag nanoparticles, the flexible and self-supporting composite nanofibrous membranes have excellent SERS properties. Serving as SERS substrates, they are extremely sensitive to the detection of 4-mercaptophenol and 4-mercaptobenzoic acid, with an enhancement factor of 10^8^. Moreover, they could be utilized to detect analytes such as pesticide thiram at a low concentration of 10^−8^ mol/L, and the substrates retain excellent Raman signals stability during the durability test of 60 days. Furthermore, the as-fabricated substrates, as a versatile SERS platform, could be used to detect bacteria of *Staphylococcus aureus* without a specific and complicated bacteria-aptamer conjugation procedure, and the detection limit is up to 10^3^ colony forming units/mL. Meanwhile, the substrates also show an excellent repeatability of SERS response for *S. aureus* organelles. Briefly, the prime novelty of this work is the fabrication of Au/Ag bimetallic synergetic enhancement substrates as SERS platform for versatile detection with high sensitivity and stability.

## 1. Introduction

Surface-enhanced Raman scattering (SERS), one of the most efficient and powerful analysis techniques, can perform ultra-sensitive, non-destructive, and trace detection of target analytes due to its unique fingerprint recognition characteristics [1,2,3]. The rational design and fabrication of SERS substrates is the key to acquiring highly sensitive SERS signals. Highly active SERS materials (especially Au and Ag nanostructures) exhibit excellent SERS activities due to their unique localized surface plasmon resonance effect, and are usually used as SERS substrates for target molecule detection [4,5]. As far as we know, when bimetallic nanomaterials are used as SERS substrates, the signal enhancement and stability are better than those of single metals [6,7]. In recent years, bimetallic nanostructures have been widely studied. For instance, Liu et al. reported a facile microbial synthesis method to fabricate Au@Ag nano-islands for quantitative SERS detection [8]. Weng et al. showed that Au nanoparticle-incorporated paper substrates prepared by the inkjet printing method can significantly improve SERS performance, and are reproduced by secondary growth of Ag nanoparticles [9]. Zhao et al. prepared a highly sensitive three-dimensional porous maize-like Ag nanoparticles/polyvinyl alcohol (PVA)@Ag SERS substrate through electrospinning and thermal evaporation technology. Due to the synergistic effect of internal and external Ag nanoparticles, it has good detection sensitivity for crystal violet and malachite green molecules [10]. Compared with the traditional preparation method, electrospinning is a versatile technique employed for fabricating nanofibrous films. The as-prepared electrospun nanofibrous mats have good flexibility, free-standing, high porosity, large specific surface area, and mechanical properties [11]. It is widely used in tissue engineering, controlled drug release, biosensing and other fields [12,13]. The electrospun nanofibers with diameters ranging from microns to nanometers are ideal templates for assembling SERS active nanoparticles. 

Recently, there have been some reports on the preparation methods of electrospun nanofibers containing metal nanostructures. One method is direct blending, in which SERS active metal nanostructures are embedded into the polymer matrix. For example, in our previous work, we employed the method of direct blending spinning to prepare PVA nanofibers incorporated with Ag nanoparticles or Ag nanowires, and verified the SERS and antibacterial activities [14,15]. Although the direct blending method is easy to conduct, due to the influence of the high voltage electric field, the morphology and size of noble metal nanoparticles are difficult to control, and they can easily to aggregate and deform. Another method is to assemble active metal nanostructures on the external surface of nanofibers. For example, in order to realize the multifunctional application of electrospun nanofibrous mats, we prepared Ag@TiO_2_ and Ag@polyacrylonitrile electrospun substrates loaded with Ag nanoparticles. The as-obtained substrates were proved not only for bacteria label-free SERS detection, but also excellent antibacterial activity [16,17]. However, Ag nanoparticles are mainly attached to the substrate through electrostatic adsorption. The interaction between the active nanoparticles and the supporting materials is weak and unstable, which impairs the SERS performance of the substrate to some extent. Hence, the questions of how to control the uniform distribution of nanoparticles on the fibers and combine nanoparticles with fibers firmly through effective chemical bonding are still challenges in this field. In addition, in the current work of noble metal/electrospun fiber composites, the noble metals are generally loaded separately inside or outside of the fibers. There are few reports on the loading of metal nanometals both inside and outside the fibers simultaneously, although these nanostructures will have excellent synergistic properties, and the related loading methods still need to be further studied. So, the related preparation method is well worth exploring.

As is well known, inorganic materials (especially silica nanofibers) have received extensive attention due to their good mechanical strength, large specific surface area, low thermal conductivity, good biocompatibility, and safety after calcination [18,19]. The superior performance of silica nanofibrous membranes makes them suitable for high heat-resistant materials, filter materials, biomedical tissue engineering, dye-sensitized solar cells, and so on [20,21]. However, few efforts have been made to investigate the application of flexible silicon nanofibers in SERS. To the best of our knowledge, there are only two reports on this filed up to now. Tang et al. employed electrospinning and pyrolysis to successfully synthesize silica nanofibrous membranes containing Ag nanoparticles, which showed good SERS activity for enrofloxacin [22]. Our group prepared Ag@polydopamine@SiO_2_ nanofiber mats through electrospinning and surface chemical modification for the label-free SERS detection of bacteria [23]. However, the above studies only reported the preparation of SiO_2_ nanofibers loaded with single noble metal nanoparticles for SERS detection, and the analytes are specific. Therefore, the preparation of bimetallic synergistically enhanced SiO_2_ electrospun nanofibrous membranes serving as SERS substrates suitable for the detection of multiple analytes is worthy of in-depth study. In addition to the above-mentioned inherent characteristics of electrospun nanofibers, SiO_2_ nanofibers as SERS substrates have the advantages of no Raman interference peaks and good resistance to laser heating. Thus, it is considered to be an excellent candidate for SERS substrates. It is worth noting that in the preparation process of SiO_2_ electrospun nanofibers, the choice of template is very important. So far, the most commonly used template is polyvinyl pyrrolidone (PVP). On the one hand, PVP can be dissolved directly in organic solvents or water, and the choice of solvents is relatively wide. On the other hand, the addition of PVP can not only increase the viscosity of the spinning solution, but also guide the formation of SiO_2_ formed in the sol-gel reaction.

Based on the above background, we propose herein a route for the fabrication of Au/Ag bimetallic flexible SERS substrate with high sensitivity, stability and good repeatability for rapid trace detection of a variety of analytes. First of all, SiO_2_@Au nanofibers were fabricated via a two-step (electrospinning and calcination) method. Then, the as-prepared SiO_2_@Au nanofibers were immersed into a solution of tannic acid (TA) and 3-aminopropyltriethoxysilane (APTES) for surface modification through the Michael addition reaction. Lastly, the Au/Ag bimetallic nanoparticle-functionalized SiO_2_ composite nanofibers (Ag@T-A@SiO_2_@Au) were obtained by in situ reduction of AgNO_3_ with TA as a reducing agent. The morphologies and structures of the composite nanofibers were intensively characterized by various techniques including scanning electron microscopy (SEM), transmission electron microscopy (TEM), X-ray powder diffraction (XRD), thermogravimetric analysis (TGA), energy-dispersive X-ray spectroscopy (EDS), and X-ray photoelectron spectroscopy (XPS). SERS activities of Ag@T-A@SiO_2_@Au composite nanofibrous membranes were assessed by using various analytes such as small molecule probes, pesticides, and macromolecules of germs. In a word, the main innovation of this paper is the provision of Ag@T-A@SiO_2_@Au nanofibrous SERS substrates with synergistic enhancement by bimetal nanoparticles via a combined process of electrospinning and surface chemical modification, which exhibit high sensitivity, stability, good repeatability, and versatility for multiple analytes’ SERS detection.

## 2. Materials and Methods 

### 2.1. Reagents

TA, APTES, PVP (Mw = 1,300,000), Tris (hydroxymethyl)-aminomethane (Tris), 4-mercaptophenol (4-MPh), and 4-mercaptobenzoic acid (4-MBA) were obtained from Aladdin Biological Technology Co., Ltd. (Shanghai, China). Tetraethylorthosilicate (TEOS), hydrochloric acid (HCl), dimethyl sulfoxide (DMSO), N, N-dimethylformamide (DMF), and absolute ethanol (EtOH) were purchased from Kemiou Chemical Reagent Co., Ltd. (Tianjin, China). Silver nitrate (AgNO_3_) was supplied by Sinopharm Chemical Reagent Co., Ltd. (Beijing, China). Chlorauric acid (HAuCl_4_∙3H_2_O) was purchased from Dongguan Sinopharm Group Co., Ltd. (Dongguan, Guangdong, China). All the chemical reagents were analytical grade (AR) and used as received. Nutrient Agar and broth medium were obtained from Beijing Aoboxing Biotechnology Corporation Biotechnology Co., Ltd. (Beijing, China). Regarding strains used in the laboratory, Gram-positive bacteria of *Staphylococcus aureus* (*S. aureus*, ATCC 6538) were purchased from Huankai Microbiotechnology Co., Ltd. (Guangzhou, China).

### 2.2. Instruments and Characterization

The setup of electrospinning device was described in our reported work [23]. SEM images were obtained by using a scanning electron microscope (FEI Nova NanoSEM 450, Thermo Fisher Scientific, Waltham, MA, USA), at acceleration voltage of 30 kV, a working distance of 5 mm, and a spot size of 3.0 nm. EDS was acquired by an energy spectrum accessory (X-MaxN, OXFORD Instruments, Abingdon, Oxon, UK) on the scanning electron microscope. TEM images were recorded by a transmission electron microscope (JEM-2100, JEOL, Ltd., Akishima, Japan) with an accelerating voltage of 200 KV. XRD patterns were acquired by using an X-ray diffractometer (BRUKER D8-ADVANCE, Bruker Co., Karlsruhe, Germany) with Cu Kα (λ = 1.5418 Å) radiation, a generator voltage of 40 kV, and a current of 40 mA. TGA data were obtained on a thermal analyzer (TAQ600, TA Instruments Inc, New Castle, DE, USA) in the temperature range of 25–800 °C with a heating rate of 10 °C/min under N_2_ atmosphere. XPS measurements were performed by an X-ray photoelectron spectrometer (ESCALAB 250XI, Thermo Fisher Scientific, Waltham, MA, USA). Raman spectra were recorded on a laser confocal Raman spectrometer (Renishaw inVia, Reniseau, UK). The vacuum tube high temperature sintering furnace (OTF-1200X) was supplied from Kejing Materials Technology Co., Ltd. (Hefei, Anhui, China). An atomic force microscope (AFM, Dimension Icon, Brook, Germany) was used to measure the surface roughness changes of the nanofibrous membranes before and after surface modification.

### 2.3. Preparation of SiO_2_@Au Nanofibers

Firstly, 0.60 g PVP was added to the mixed solution of 3.25 g DMF and 0.65 g DMSO, and stirred for 3 h. Next, 0.80 g TEOS was slowly added to 0.30 g HCl (3 drops of 6.0 mol/L hydrochloric acid added to 25 mL distilled water) and 0.20 g ethanol solution, and stirred at room temperature for 10 h. Then, the mixed solution of PVP and TEOS was magnetically stirred for 3 h to obtain a homogeneous electrospun solution. Subsequently, different qualities of HAuCl_4_∙3H_2_O (10 mg, 20 mg, and 30 mg) were added to the spun solution, and stirring was continued for 3 h. Finally, a yellow transparent precursor spun solution was obtained. The precursor spun solution was then placed in a 10 mL plastic syringe equipped with a 22-gauge stainless steel needle for spinning. The electric field voltage was 16 kV, the feed rate was 0.8 mL/h, and the acceptance distance was 15 cm. In order to obtain SiO_2_@Au nanofibers, the precursor nanofibers were placed in a tube furnace under air atmosphere at 200 °C for 2 h with a temperature rising rate of 1.5 °C/min. Subsequently, the sample was calcined further to 600 °C for 3 h at a rate of 3 °C/min. Lastly, the product was naturally cooled to room temperature. As a result, red SiO_2_@Au nanofibrous membranes with different Au contents were obtained (hereinafter noted as SiO_2_@Au-10, SiO_2_@Au-20, SiO_2_@Au-30, respectively). 

### 2.4. Preparation of T-A@SiO_2_@Au Nanofibers

Firstly, 5.0 mL ethanol and 50 mg APTES were added to the Tris-HCl solution (25 mL, pH = 8.5) containing 50 mg of TA to obtain a TA-APTES mixed solution. Then, 10 mg SiO_2_@Au-20 nanofibers were immersed in the fresh prepared TA-APTES mixture solution for 12 h at room temperature. After the reaction was completed, the modified nanofibers were washed thoroughly with deionized water, and dried in a vacuum drying cabinet at 40 °C for 12 h. The obtained product was labeled as T-A@SiO_2_@Au-20 nanofibers. 

### 2.5. Preparation of Ag@T-A@SiO_2_@Au Nanofibers

The as-prepared T-A@SiO_2_@Au-20 electrospun nanofibrous membranes were immersed in the newly prepared AgNO_3_ solution with a concentration of 0.10 mol/L for 30 min, and reduced in situ to generate Ag nanoparticles. Then, the nanofibrous membranes were washed with distilled water multiple times and dried in vacuum for 12 h to obtain Ag@T-A@SiO_2_-Au-20 nanofibers. 

### 2.6. SERS Measurement for Small Molecules

In the SERS experiment, small molecule probes of 4-MBA, 4-MPh and pesticide of thiram were applied as target analyte molecules. Firstly, the as-prepared Ag@T-A@SiO_2_-Au-20 nanofibrous substrates were immersed in ethanol solutions of target analyte molecules with different concentrations for 2 h, respectively. Secondly, the nanofibrous substrates samples were washed three times with ethanol and then dried in air. Lastly, the corresponding SERS spectra were recorded with a 633 nm He-Ne laser. The detection wavelength range of Raman signals is 300–1800 cm^−1^, the eyepiece multiple is 50×, the laser exposure time is 10 s, and the power is 0.5 mW. 

The enhancement factor (EF) of Ag@T-A@SiO_2_-Au-20 nanofibrous substrates was estimated according to the following equation [24]:

*EF* = (*I_SERS_* × *C_NR_*)/(*I_NR_* × *C_SERS_*) (1)

where *I_SERS_* and *I_NR_* are the signal intensities of SERS and normal Raman spectra, respectively; while *C_SERS_* and *C_NR_* are the concentration of the analyte molecule detected by the Raman instrument, respectively.

In order to verify the SERS detection stability of the as-prepared electrospun nanofibrous membranes substrates, the durability test was performed by continuously washing the substrate with absolute ethanol after adsorbing the analytes. The procedure is as follows: firstly, the nanofibrous membranes adsorbing 10^−5^ mol/L probe molecules were detected for obtain SERS spectra. After that, the membranes were washed with 10 mL absolute ethanol thoroughly. Subsequently, they were dried in air for the next SERS detection. This washing recycle procedure was repeated 5 times.

### 2.7. SERS Detection of Bacteria

Six milliliters of the overnight cultivated bacterial suspension containing 10^9^ colony-forming unit (cfu)/mL *S. aureus* was collected at 6000 rpm for 5 min. Subsequently, the bacterial precipitate obtained after centrifugation was washed 3 times with 0.9% NaCl solution, and then dispersed in 200 μL 0.9% NaCl solution. Finally, 15 μL of bacterial suspension was dropped on the surface of the Ag@T-A@SiO_2_-Au-20 nanofibrous membranes, and dried in air for 15 min. The SERS detection was performed with a laser with a wavelength of 785 nm. In the Raman test, the objective lens is 100×, the exposure time is 10 s, the laser power is 0.5 mW, and the acquisition range is 300–1800 cm^−1^.

**Figure 1:**
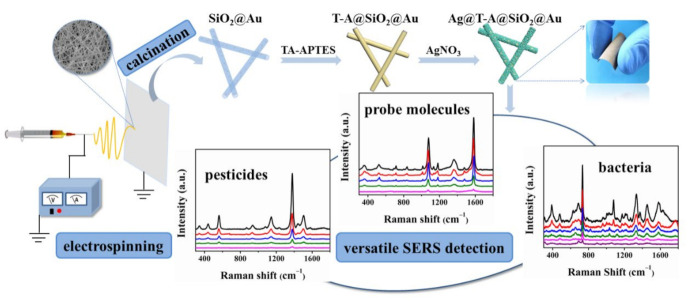
Schematic illustration of fabrication and surface-enhanced Raman scattering (SERS) activity for Ag@T-A@SiO_2_-Au nanofibrous substrates.

Briefly, we fabricated Ag@T-A@SiO_2_-Au nanofibrous membranes by combination with electrospinning and surface chemical modification processes, and investigated its SERS activities. The whole research route can be summarized as a schematic illustration, as shown in Figure 1. 

## 3. Results and Discussion

### 3.1. Preparation and Characterization of Ag@T-A@SiO_2_@Au Nanofibers 

**Figure 2:**
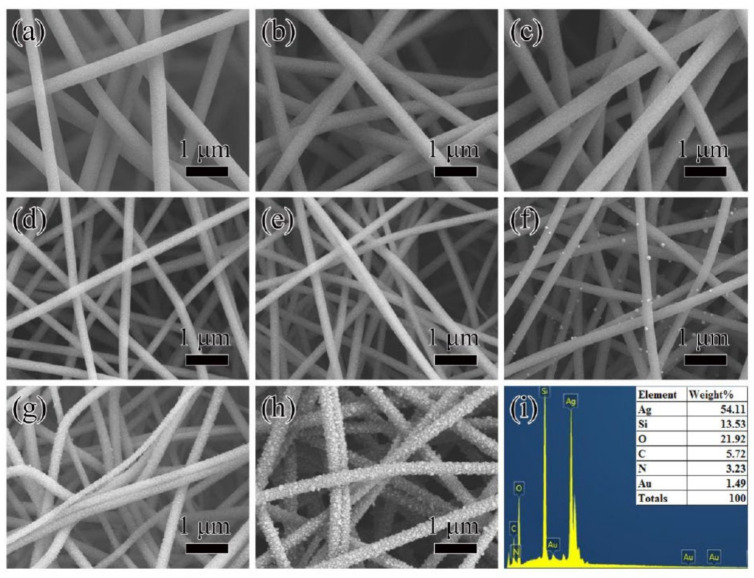
SEM images for nanofibers of SiO_2_@Au-10 precursor (**a**), SiO_2_@Au-20 precursor (**b**), SiO_2_@Au-30 precursor (**c**), SiO_2_@Au-10 (**d**), SiO_2_@Au-20 (**e**), SiO_2_@Au-30 (**f**), T-A@SiO_2_@Au-20 (**g**), Ag@T-A@SiO_2_@Au-20 (**h**); and EDS spectra of Ag@T-A@SiO_2_@Au-20 nanofibers (**i**).

Figure 2 shows SEM images for nanofibers of SiO_2_@Au-10 precursor (a), SiO_2_@Au-20 precursor (b), SiO_2_@Au-30 precursor (c), SiO_2_@Au-10 (d), SiO_2_@Au-20 (e), SiO_2_@Au-30 (f), T-A@SiO_2_@Au-20 (g), Ag@T-A@SiO_2_@Au-20 (h); and EDS spectra of Ag@T-A@SiO_2_@Au-20 nanofibers (i). It can be seen from Figure 2a–c that the surface of SiO_2_@Au precursor nanofibers with different Au content is smooth and flat, and the fibers are interlaced in a horizontal and vertical manner to form a three-dimensional network structure. In contrast, the SiO_2_@Au nanofibers keep the smooth, even, and uniform surface morphology of precursor nanofibers after calcination, as shown in Figure 2d–f. It is worth noting that in Figure 2d,e, no particles are observed on the surface of the nanofibers. However, it can be clearly seen from Figure 2f that nanoparticles emerged on the surface of SiO_2_@Au-30 nanofibers, accompanied by the increase in Au content. This is because the excitation electrons have a limited ability to penetrate SiO_2_ nanofibers under an acceleration voltage of 5 kV, such that Au nanoparticles embedded in the fibers cannot be imaged. Nevertheless, if too much chloroauric acid is added, some Au nanoparticles will be released from the inside of the fibers after electrospinning and calcination. Since the purpose of this study is to prepare a bimetallic nanoparticle Raman enhanced substrate, Au nanoparticles are required to be embedded in SiO_2_ nanofibers and have a high content. Therefore, it is more appropriate to select SiO_2_@Au-20 samples for the further work. Compared with Figure 2a–c and Figure 2d–f, it is found that the average diameters of nanofibers are significantly decreased after calcination. Taking the sample of SiO_2_@Au-20 as an example, it can be seen that the mean diameters of nanofibers before and after calcination are decreased from 358 ± 30 to 218 ± 29 nm, as shown in Figure S1a,b. The decrease in fibers’ diameter is due to the removal of a large amount of organic matter (such as PVP), dehydration of SiO_2_ precursor, and the decomposition of HAuCl_4_ during the calcination, leaving only Au nanoparticles embedded in SiO_2_ nanofibers, which can be furtherly proved in the XRD analysis. From Figure 2g, it can be seen that after the TA-APTES modification, some nanofibers are bent in shape and the surfaces of nanofibers are not ever smooth but change to a rough morphology. The formation of this rough coating on the surfaces of SiO_2_@Au nanofibers can be attributed to the Michael addition reaction between the oxidation product of TA and the hydrolysis product of APTES [25]. Correspondingly, it can be seen from Figure S1c that the diameter of T-A@SiO_2_@Au-20 nanofibers increased to 267 ± 31 nm. Moreover, it is seen from Figure 2h that after 30 min of immersion in 0.1 mol/L AgNO_3_ solution, a large number of nanoparticles formed and were deposited on the surfaces of T-A@SiO_2_@Au-20 fibers, which causes the nanofibers’ diameter to increase to 355 ± 33 nm, as shown in Figure S1d. Obviously, Ag nanoparticles are coated on the surface of nanofibers. It is supposed that the formation process is as follows: the modification layer of TA-APTES contains a large number of hydroxyl and amino groups, which can chelate Ag^+^, and the hydroxyl group in TA can reduce Ag^+^ to Ag nanoparticles in situ. It also can be found from Figure 2h that these Ag nanoparticles cover almost the entire surface of the fibers uniformly and densely, and the gaps between adjacent Ag nanoparticles are in the nanometer scale, which facilitates the formation of SERS hot spots, and improves the SERS sensitivity. As a micro-domain element analysis tool, EDS spectroscopy can be applied to confirm the elemental composition of the composite nanofibers. Figure 2i verifies the existence of Si, O, C, N, Au, and Ag elements in Ag@T-A@SiO_2_@Au-20 nanofibers. It is worth pointing out that the percentage of Ag atoms is rather high, which helps to improve its SERS effect.

**Figure 3:**
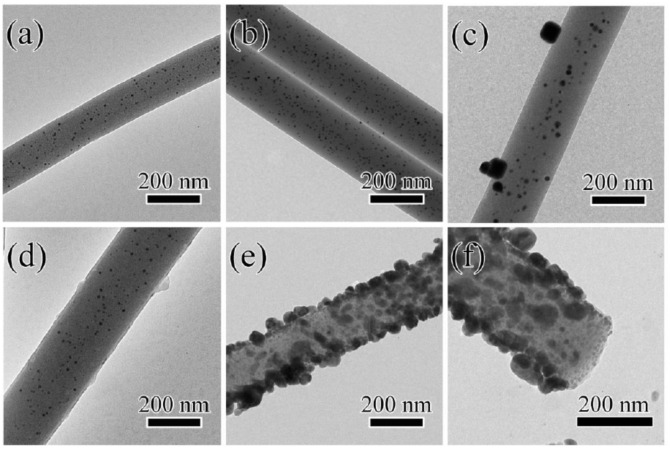
TEM images of SiO_2_@Au-10 (**a**), SiO_2_@Au-20 (**b**), SiO_2_@Au-30 (**c**), T-A@SiO_2_@Au-20 (**d**), Ag@T-A@SiO_2_@Au-20 (**e**) nanofibers, and the local enlarged cross-sectional view of Ag@T-A@SiO_2_@Au-20 (**f**).

In order to more clearly reveal the microstructure of the composite nanofibers and confirm the incorporation of Au nanoparticles inside the fibers, the Ag@T-A@SiO_2_@Au nanofibers were characterized by TEM. Figure 3 shows TEM images of SiO_2_@Au-10 (a), SiO_2_@Au-20 (b), SiO_2_@Au-30 (c), T-A@SiO_2_@Au-20 (d), Ag@T-A@SiO_2_@Au-20 (e) nanofibers, and the local enlarged cross-sectional view of Ag@T-A@SiO_2_@Au-20 (e). As can be seen from Figure 3a, when the added amount of HAuCl_4_ is 10 mg, the surface of SiO_2_@Au-10 nanofibers is smooth and flat, and the distribution of Au nanoparticles inside SiO_2_ is relatively scattered, while the average particle size of Au nanoparticles is about 6.1 nm, as shown in Figure S2a. When the added amount of HAuCl_4_ is increased to 20 mg, it can be seen from Figure 3b that a large number of Au nanoparticles are evenly distributed inside SiO_2_ nanofibers, and these Au nanoparticles are spherical with an average particle size of 8.2 nm, as shown in Figure S2b. However, it can be seen from Figure 3c that Au nanoparticles are formed inside and outside of SiO_2_ nanofibers when the added amount of HAuCl_4_ is further increased to 30 mg, and the agglomeration of Au nanoparticles is obviously observed. Correspondingly, the size of the Au nanoparticles increased to 15.7 nm, as shown in Figure S2c. Therefore, in this study, the SiO_2_@Au-20 nanofibers with a large load density and uniform distribution of Au nanoparticles were selected for the next step of sample preparation. It can be seen from Figure 3d that after TA-APTES modification, a thin layer of rough coating appears on the surface of the nanofibers and some aggregates also occurred, which is consistent with the SEM results. It can be found from Figure 3e that Ag nanoparticles are formed on the surface of T-A@SiO_2_@Au-20 nanofibers after it is immersed in AgNO_3_ solution, and the mean particle size of Ag nanoparticles is about 33.2 nm, as shown in Figure S2d. Moreover, the dense and uniform distribution of Au nanoparticles inside the nanofibers and the successful decoration of Ag nanoparticles on the fiber surface can be clearly distinguished from the local enlarged cross-section view, as shown in Figure 3f.

In order to further confirm that the surface of nanofibers was successfully modified, AFM was used to characterize the surface roughness changes of SiO_2_ composite nanofibrous membranes before and after modification. Figure S3 shows the three-dimensional and two-dimensional AFM images of SiO_2_@Au-20 and Ag@T-A@SiO_2_@Au-20 electrospun nanofibrous membranes. It can be seen from Figure S3a,c that the surface of the SiO_2_@Au-20 nanofibers is smooth and flat, and these nanofibers randomly crisscross to form a three-dimensional network structure. However, after the surface modification, it can be clearly seen from Figure S3b,d that the fiber surface is no longer smooth, but coarse particles appear. In addition, it can be seen from Figure S3 that the arithmetic mean deviation of the profile (*Ra*) changed from 185 before modification to 220 after treatment, indicating that the surface roughness of the nanofibrous membrane increased significantly.

Based on the above SEM, TEM, and AFM observation, the morphology and structure of Au and Ag bimetallic nanoparticles decorated inside and outside of SiO_2_ nanofibers is clearly revealed. It is supposed that the as-prepared Ag@T-A@SiO_2_@Au composite nanofibers would exhibit excellent SERS activities. This is due to the fact that the internal Au nanoparticles possess high stability during SERS detection, and external Ag nanoparticles have a large number of SERS “hot spots”, and the bimetallic nanoparticles will form a synergistic enhancement effect.

**Figure 4:**
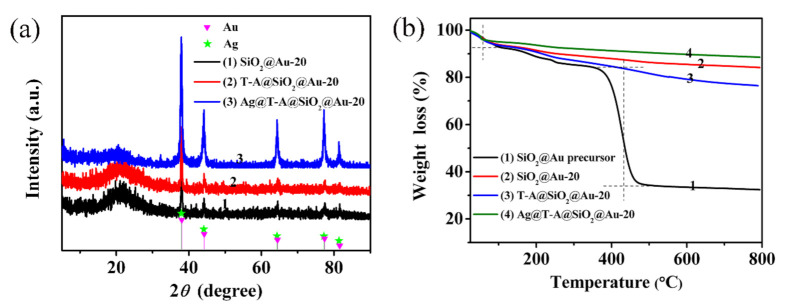
XRD patterns (**a**) of SiO_2_@Au-20, T-A@SiO_2_@Au-20 and Ag@T-A@SiO_2_@Au-20 nanofibers; TGA curves (**b**) of SiO_2_@Au-20 precursor, SiO_2_@Au-20, T-A@SiO_2_@Au-20 and Ag@T-A@SiO_2_@Au-20 nanofibers.

The crystal structure of Au and Ag nanoparticles was determined by XRD. Figure 4a shows the diffraction patterns of SiO_2_@Au-20, T-A@SiO_2_@Au-20 and Ag@T-A@SiO_2_@Au-20 nanofibers, respectively. As seen from Figure 4a, SiO_2_@Au-20 nanofibers exhibit diffraction peaks at 23.1°, 38.1°, 44.3°, 64.3°, 77.4°, and 81.5°. Among of them, the diffraction peak at 23.1° belongs to the amorphous SiO_2_ phase, while the peaks at 2θ = 38.1°, 44.3°, 64.3°, 77.4°, and 81.5°are assigned to the (111), (200), (220), (311) and (222) crystal planes of face-centered cubic (fcc) Au (JCPDS, No. 04-0784), respectively [7,23]. After the TA-APTES modification, the diffraction peaks of T-A@SiO_2_@Au-20 nanofibers do not show significant changes, indicating that the modification TA-APTES on the nanofiber’s surfaces has no effect on the crystal structure of SiO_2_@Au nanofibers. However, when Ag nanoparticles were further decorated on the nanofiber’s surface, the XRD spectrum of the Ag@T-A@SiO_2_@Au-20 nanofibers show similar diffraction patterns consistent with the SiO_2_@Au-20 nanofibers, except for the obvious enhancement of the peak’s intensity. This is because the diffraction peak positions of fcc Ag (JCPDS, No. 04-0783) [26] are almost as same as those of fcc Au. Meanwhile, the dense deposition of Ag nanoparticles on the surface of nanofibers increases the intensity of diffraction peaks.

The thermal decomposition process of nanofibrous membranes were detected by the technique of TGA. Figure 4b shows TGA curves of SiO_2_@Au-20 precursor, SiO_2_@Au-20, T-A@SiO_2_@Au-20 and Ag@T-A@SiO_2_@Au-20 nanofibers. For the sample of SiO_2_@Au-20 precursor nanofibers, there are three stages of decomposition. The first step occurs from 25 to 100 °C, and the weight loss is about 7.1%. This is due to the volatilization of adsorbed water and the residual solvent in the nanofiber’s precursor. The second step occurs from 100 to 350 °C; the sample loses 7.7% of its mass, which can be attributed to the dehydration of TEOS, the decomposition of PVP side chains and HAuCl_4_. The last step with a significant mass loss occurs from 350 to 500 °C—the weight loss is about 50.1%, which is assigned to the decomposition of the PVP skeleton and the condensation reaction of TEOS [23,27]. Note that there is almost no mass loss for the SiO_2_@Au precursor nanofibers after 500 °C, suggesting the complete formation of SiO_2_@Au nanofibers. Based on the result obtained above, a high-temperature calcination of 600 °C was selected to obtain SiO_2_@Au nanofibers from the as-prepared electrospun SiO_2_@Au-20 precursor nanofibers in this study. Comparing the TGA curves of SiO_2_@Au-20 and T-A@SiO_2_@Au-20 nanofibers, it can be found that the total mass loss of SiO_2_@Au-20 and T-A@SiO_2_@Au-20 nanofibers is 15.8% and 23.5%, respectively. The mass loss difference value is about 7.7%, which is obviously due to the thermo decomposition of the TA-APTES modification layer. In addition, it can be seen from the TGA curve of Ag@T-A@SiO_2_@Au-20 nanofibers that the Ag nanoparticles decorated on the surface of the nanofibers do not decompose, and the total weight loss is about 11.5%, even when the temperature is raised to 800 °C. Generally, the as-prepared Ag@T-A@SiO_2_@Au nanofiber membranes in this work exhibit high thermal stability. Good thermal stability is one of the key advantages of SERS substrates, since they can endure laser radiation.

**Figure 5:**
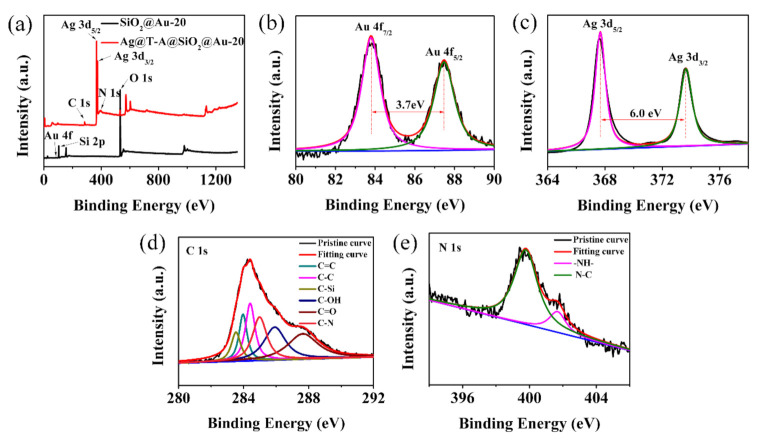
XPS full spectra of SiO_2_@Au-20 and Ag@T-A@SiO_2_@Au-20 nanofibers (**a**), the divided peak spectra of Au (**b**), Ag (**c**), C (**d**), and N (**e**) elements.

In addition, the XPS analysis method was applied to further explore the surface chemical structure of electrospun nanofibrous membranes for verification of the modification of TA-APTES and Ag nanoparticles. Figure 5 shows the XPS full spectra (a) of SiO_2_@Au-20 and Ag@T-A@SiO_2_@Au-20 nanofibers, and the divided peak spectra of Au (b), Ag (c), C (d), N (e). Comparing the two XPS full spectra in Figure 5a, it can be clearly observed that after modification with TA-APTES and Ag nanoparticles, apart from Si, O, and Au, there are new signal peaks of C, N, and Ag in Ag@T-A@SiO_2_@Au-20 nanofibers, which is consistent with the chemical composition of the composite nanofibers. It is worth noting that the signal peak of Au is very weak in the spectrum of nanofibers. The reason may be the relatively low content of Au atoms in the nanofibers, as shown in Figure 2i above. In addition, XPS is a surface analysis tool, and Au nanoparticles are incorporated into the fiber, which affects its peak intensity to a certain extent. From the high-resolution XPS spectrum of the Au 4f orbital region (Figure 5b), it can be seen that the binding energy peaks of Au 4f_7/2_ and Au 4f_5/2_ are 83.4 and 87.1 eV, respectively. The splitting energy of the 4f doublet is 3.7 eV, which indicates the existence metallic state of Au^0^ [28]. Meanwhile, in the spectrum of Ag 3d, as shown in Figure 5c, the typical peaks of Ag 3d_5/2_ and Ag 3d_3/2_ appear at 368.2 and 374.2 eV, with a spin-orbit separation of 6.0 eV, which can be attributed to the metallic state of Ag, implying that the metallic state of Ag formed on the surface of the SiO_2_ nanofibers [29]. Figure 5d shows the sub-peaks corresponding to C 1s. There are six peaks at 283.6, 284.1, 284.5, 285.1, 286.2 and 287.8 eV in the C 1s whole spectrum, which are attributed to C–Si, C = C, C−C, C−N, C−OH and C = O bonds, respectively [30]. In the N 1s spectrum of Figure 5e, the peaks of 399.5 and 401.7 eV correspond to the N−C, −NH− bond, respectively [25,31]. The appearance of C−N and −NH− bonds further confirms that the Michael addition reaction between TA and APTES does occur. This reaction can produce a rough surface coating similar to a binder on the fiber surface. The coating can play the role of bridging, and can firmly bond the SiO_2_@Au substrate with the Ag nanoparticles, so that the substrate has high detection sensitivity and excellent stability during the SERS detection process. Combined with the above results of the SEM, TEM and XRD analysis, the XPS spectra confirm once again the chemical structure of Au/Ag bimetallic nanoparticles modified SiO_2_ nanofibers.

**Figure 6:**
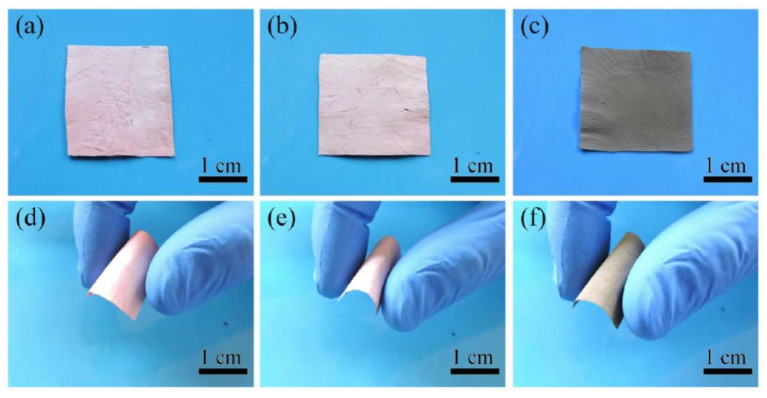
Photographs of nanofibrous membranes of SiO_2_@Au-20 (**a**,**d**), T-A@SiO_2_@Au-20 (**b**,**e**), Ag@T-A@SiO_2_@Au-20 (**c**,**f**).

Figure 6 shows the photographs of nanofibrous membranes SiO_2_@Au-20 (a,d), T-A@SiO_2_@Au-20 (b,e), Ag@T-A@SiO_2_@Au-20 (c,f) before and after manual folding. From Figure 6a–c, it can be clearly seen that after the modification by TA-APTES and Ag nanoparticles, the color of SiO_2_@Au membranes changes from the original pink to light red, and then to black, indicating that TA-APTES and Ag nanoparticles are deposited on the surface of the nanofibrous membranes. Simultaneously, a flexibility experiment was performed on the as-prepared membranes by manual bending, as displayed in Figure 6d–f. It can be clearly seen that the SiO_2_@Au nanofibrous membranes can be bent 180° without breaking after being calcined at a high temperature of 600 °C. Furthermore, the samples modified by TA-APTES and Ag nanoparticles also maintain the good flexibility of SiO_2_@Au nanofibers. This good flexibility ensures that the electrospun layered nanofibrous membrane is not easy to break, combined with the large specific surface area and porosity feature, meaning the substrates can collect trace amounts of target analyte molecules effectively, which is a critical factor which has been neglected in practical SERS application [32,33].

### 3.2. SERS Activities for Small Molecules

**Figure 7:**
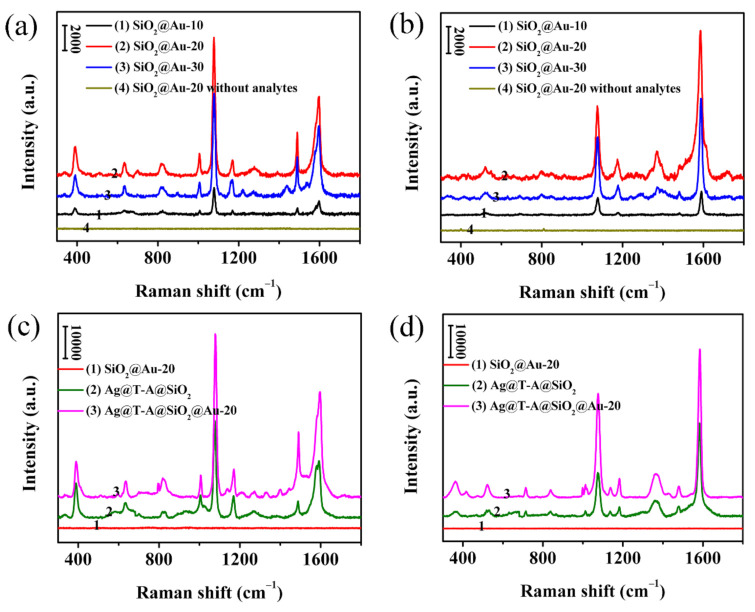
SERS spectra of 10^−1^ mol/L 4-MPh (**a**) and 4-MBA (**b**) adsorbed on SiO_2_@Au nanofibrous membranes; SERS spectra of 10^−5^ mol/L 4-MPh (**c**) and 4-MBA (**d**) detected on SiO_2_@Au-20, Ag@T-A@SiO_2_, Ag@T-A@SiO_2_@Au-20 nanofibrous membranes, respectively.

To evaluate the SERS activity, comparative experiments were conducted by recording SERS spectra of 4-MPh and 4-MBA adsorbed on SiO_2_@Au, Ag@T-A@SiO_2_, and Ag@T-A@SiO_2_@Au nanofibers, as shown in Figure 7. Figure 7a presents the SERS spectra of 4-MPh (10^−1^ mol/L) molecules collected on SiO_2_@Au nanofibers with different HAuCl_4_ contents. It can be seen from Figure 7a that 4-MPh molecules adsorbed on the SiO_2_@Au nanofibrous membranes can all produce obvious SERS peaks. The SERS peaks at 390, 638, 824, 1007, 1073, 1490 and 1596 cm^−1^ correspond to the stretching and bending vibrations of the groups in the 4-MPh molecule [34]. By the comparison of the spectra for different samples in Figure 7a, it can be found that the 4-MPh SERS signals collected by the SiO_2_@Au-20 sample are the strongest. The best SERS effect can be attributed to the uniform distribution and high load density of Au nanoparticles in the fibers, as revealed by the above TEM analysis. The probes molecules can diffuse into the fibers and in full contact with Au nanoparticles to produce more hot spots, which is conducive to the provision of SERS signals. In addition, in order to verify that the as-prepared nanofibrous membrane substrate itself has no characteristic Raman peaks that interfere with SERS detection, Raman analysis of neat films without analytes was conducted. It can be seen from Figure 7a,b that there is no Raman signal from SiO_2_@Au-20 membranes without analytes. This feature helps in the simplification of SERS detection. Furthermore, to investigate the SERS enhancement properties of the modified nanofibers, 4-MPh molecules at a concentration of 10^−5^ mol/L, were detected by substrates of SiO_2_@Au-20, Ag@T-A@SiO_2_ and Ag@T-A@SiO_2_@Au-20, as shown in Figure 7c. Comparing the SERS spectra of three different samples in Figure 7c, it is found that the 4-MPh SERS peak intensity of Ag@T-A@SiO_2_@Au-20 composite nanofibrous membranes substrate is the highest, indicating that this sample has the most significant Raman enhancement effect on 4-MPh. The reason can be attributed to the fact that Au and Ag electromagnetic field synergistic enhancement in nanofibers can provide abundant SESR hot spots. The laser can not only irradiate Ag nanoparticles on the fibers’ surface, but also pass through the surface SiO_2_ nanofibers and come into contact with the embedded Au nanoparticles, thus causing plasmon resonance [10]. Similarly, Figure 7b,d show the SERS detection results of the as-prepared samples on another probe molecule, i.e., 4-MBA. It can be perceived from Figure 7b that the SERS peaks centered at 523, 1080, 1186, and 1587 cm^−1^ are attributed to the characteristic Raman absorption of 4-MBA adsorbed on the fibrous samples [35], and the SERS signal intensity of the SiO_2_@Au-20 nanofibers is significantly higher than the other two samples. In addition, it can also be found that Ag@T-A@SiO_2_@Au-20 nanofibers show the strongest SERS effect in three different samples, as shown in Figure 7d, which is consistent with the results of 4-MPh. Combining the morphology and structure analysis with SERS results, it is demonstrated that Ag@T-A@SiO_2_@Au-20 nanofibrous membranes possess the optimal SERS activities, and these membranes were selected for the detection of other analytes in the coming experiment.

**Figure 8:**
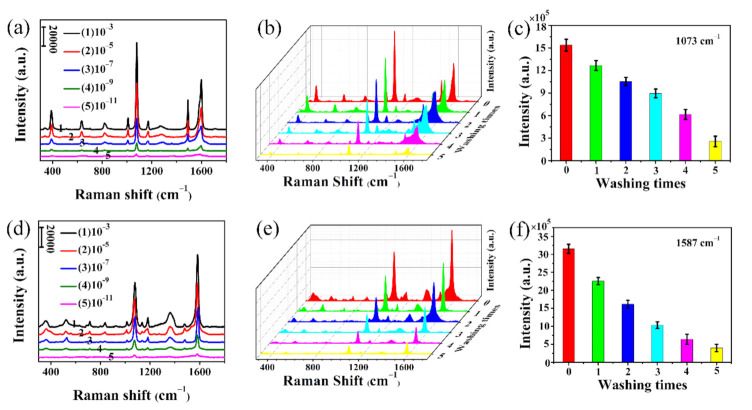
SERS spectra of Ag@T-A@SiO_2_@Au-20 substrates measured with 10^−3^–10^−11^ mol/L 4-MPh (**a**) and 4-MBA (**d**); SERS spectra of Ag@T-A@SiO_2_@Au-20 substrates measured with 10^−5^ mol/L 4-MPh (**b**) and 4-MBA (**e**) for 5 consecutive cleaning; the peak intensity at 1073 cm^−1^ for 4-MPh (**c**) and 1587 cm^−1^ for 4-MBA (**f**) during SERS washing resistance test.

In order to further investigate the SERS performance of Ag@T-A@SiO_2_@Au-20 nanofibrous substrates, the Raman spectra of 4-MPh molecules with different concentrations (from 10^−3^ to 10^−11^ mol/L) on the substrate are detected, as shown in Figure 8a. It is found that the peak intensities decrease with the decay of the 4-MPh concentration. However, a well-resolved Raman spectrum can still be clearly observed, even when the concentration is as low as 10^−11^ mol/L. This means that Ag@T-A@SiO_2_@Au-20 electrospun nanofibrous membranes as the SERS substrate have a very high detection sensitivity for 4-MPh. Meanwhile, in order to verify the applicability of the substrate to different probe molecules, similar detection was performed on 4-MBA molecules. For this molecule, except for the fact that the characteristic peak position changed, the other SERS detection results are similar to 4-MPh. It can be found from Figure 8d that the peak intensity is related to the concentration of the probe molecules, and the detection limit for 4-MBA also reaches 10^−11^ mol/L. Additionally, the SERS EF on the Ag@T-A@SiO_2_@Au-20 nanofibrous membranes was caudated by using 4-MPh and 4-MBA as the target analytes. In this test, Ag@T-A@SiO_2_ nanofibers that adsorbed 10^−11^ mol/L probe molecules and the blank silicon wafer that adsorbed 10^−3^ mol/L probe molecules are compared. The EFs of the nanofibrous platform are calculated as 5.4 × 10^8^ for 4-MPh and 2.3 × 10^8^ for 4-MBA, respectively (the detail data are shown in Table S1). Compared with the different electrospun SERS substrates reported by other researchers, as shown in Table S2, the as-prepared Ag@T-A@SiO_2_@Au-20 nanofibrous substrates possess a higher EF. Obviously, this can be attributed to the synergistic Raman enhancement effect of the bimetallic nanoparticles. The above-mentioned results adequately prove that the as-prepared electrospun nanofibrous membranes have the capability to act as an excellent SERS substrate, and can realize trace detection for small probe molecules, which are higher than our previous reports [16,17] and other test results of similar Ag composite nanostructures [36].

In order to further study the stability of SERS detection for the as-prepared Ag@T-A@SiO_2_@Au-20 nanofibrous membranes, a durability test was performed through consecutively washing the substrates by absolute ethanol after the analytes were adsorbed. Figure 8b,e show the SERS spectra for 4-MPh and 4-MBA (10^−5^ mol/L) versus cleaning times. Correspondingly, the intensity of the strongest peak in 4-MPh and 4-MBA (i.e., 1073 and 1587 cm^−1^) changes with washing time, as shown in Figure 8c,f, respectively. It is found that the characteristic Raman peak intensity decreases slowly with the increase in washing time. However, even after five consecutive washings with ethanol, the clearly visible characteristic Raman peak can still be detected. This proves that Ag@T-A@SiO_2_@Au-20 nanofibrous membranes provide high SERS detection stability, which is due to the incorporation of Au nanoparticles into SiO_2_ nanofibers and firm binding of Ag nanoparticles through chemical bonding by TA-APTES.

**Figure 9:**
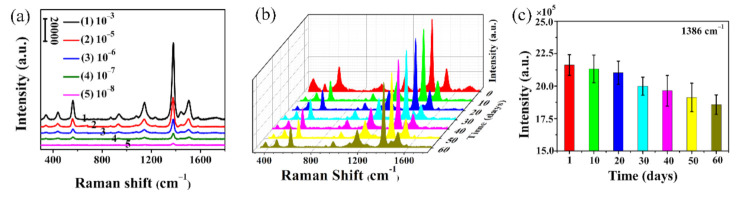
SERS spectra of thiram with different concentrations adsorbed on Ag@T-A@SiO_2_@Au-20 nanofibrous membranes (**a**), SERS stability spectra of 10^−5^ mol/L thiram on Ag@T-A@SiO_2_@Au-20 nanofibrous membranes exposed in the atmosphere for 60 days (**b**), the peak intensity changes at 1386 cm^−1^ for thiram with standing time (**c**).

Rapid detection and identification of toxic substances in water or an aquatic environment is one of the important tasks in SERS analysis [37]. After verifying the high SERS detection sensitivity through small probe molecules, for practical applications, the as-prepared nanofibrous membranes were also used as SERS substrates for pesticide detection. Figure 9a shows the SERS spectra of thiram with concentrations increasing from 10^−8^ to 10^−3^ mol/L. From Figure 9a, it can be found that the Raman characteristic peaks of thiram appear at 560, 928, 1150, 1386 and 1514 cm^−1^, respectively, which are consistent with the results reported in the literature [38]. At the same time, within the concentration range shown in this figure, the intensity of characteristic peak at 1386 cm^−1^ was used as the quantitative basis to evaluate the SERS sensitivity. The results show that thiram can still be clearly identified at a low concentration of 10^−8^ mol/L, showing better sensitivity than other Ag nanostructured SERS substrates previously reported [1]. It is worth mentioning that this detection limit is lower than the U.S. Environmental Protection Agency’s standard requirements for the allowable minimum residue concentration of pesticides thiram [39], so it can be used for trace detection of this pesticide. For the actual application, the stability of SERS substrate is an important issue to be considered in use. The stability of the as-prepared substrate was further investigated by durability test. The nanofibrous SERS substrates prepared in the same batch were soaked in 10^−5^ mol/L thiram solution, dried and stored in air for 60 days, and SERS detection was performed every 10 days, and the results are shown in Figure 9b,c. It can be seen from Figure 9b that the peaks positions and intensities are the same. In addition, the characteristic peak intensity of thiram at 1386 cm^−1^ preserved for 60 days was analyzed. It was found that the SERS signal intensity decreased by only 14.1%, when the storage time was 60 days. This result is superior to that of the similar work [29,40]. This good detection sensitivity and stability can be attributed to the following facts. On the one hand, it is related to the molecule structure of thiram, since the S–S bond of thiram can be cleaved into two methylene residues, which can be strongly adsorbed into the three-dimensional network electrospun nanofibrous membranes [41]. On the other hand, it is well known that inner Au nanoparticles have good stability, and the external Ag nanoparticles are bonded with hydroxyl and amino groups of TA-APTES, which can prevent oxidation of metal nanoparticles. Therefore, the stable Au and Ag nanoparticles can keep their surface plasma resonance activities for a long time, so that the SERS substrates have good SERS detection stability.

### 3.3. SERS Performance for Bacteria Detection

**Figure 10:**
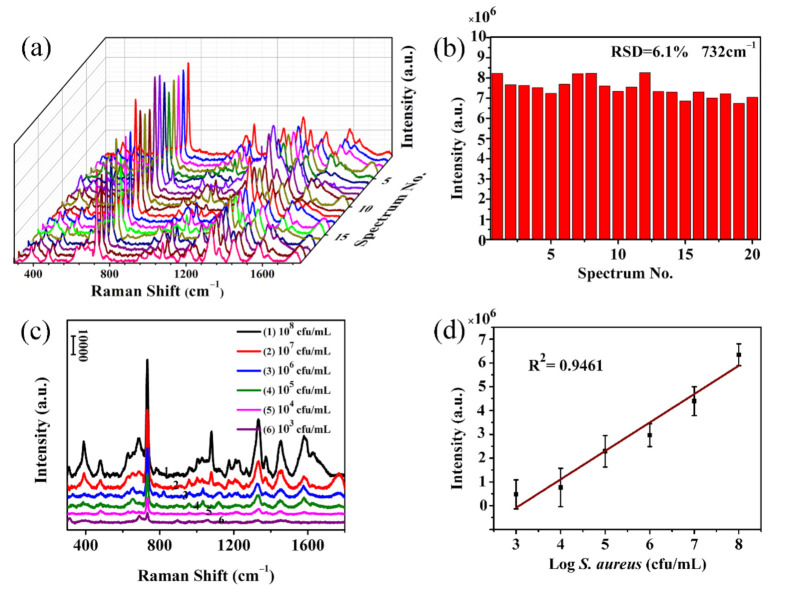
SERS spectra collected from 20 randomly selected points on Ag@T-A@SiO_2_@Au-20 substrate surfaces with a *S. aureus* concentration of 10^9^ cfu/mL (**a**), intensity distributions of characteristic Raman peak at 733 cm^−1^ from *S. aureus* SERS spectra (**b**), SERS spectra of *S. aureus* with different concentrations (**c**), the relationship plot between the peak intensity and the *S. aureus* concentration, and the fitting curve (**d**).

SERS detection of small molecule probes and pesticide thiram demonstrates that the as-prepared nanofibrous substrates have excellent detection sensitivity and good stability. Next, *S. aureus* was selected as the target strain to verify the feasibility of substrate detection of biomacromolecules. The SERS peaks of bacteria are mainly derived from proteins, polysaccharides, nucleic acids, carbohydrates and lipids in bacterial cell structure [42,43]. Therefore, through SERS spectrum matching, different bacterial structure information can be obtained to distinguish bacteria. Before SERS detection, the adsorption of bacterial strains on the electrospun nanofibrous membrane was first observed by SEM. Figure S4 shows SEM images for different magnifications of *S aureus* attached on the Ag@T-A@SiO_2_@Au-20 nanofibrous membranes. It can be seen from Figure S4 that a large number of *S. aureus* strains are adsorbed on the fibrous membranes. The reason is that the electrospun nanofibrous membranes have a relatively large specific surface area and porosity, which is beneficial to physical adsorption, and the surface modification layer of TA-APTES contains a large number of hydroxyl and amino groups that can bind to functional groups on the surface of bacterial cells. In order to verify the repeatability of the SERS substrates, 20 points were randomly selected on the Ag@T-A@SiO_2_@Au-20 nanofibrous membranes, and the SERS spectra of *S. aureus* (10^9^ cfu/mL) were measured under the same conditions, as shown in Figure 10a. It can be found from this figure that all SERS spectra show a high degree of uniformity in both the position and intensity of peaks. There are several main SERS peaks at 733, 1327, 1444 and 1576 cm^−1^, corresponding to the vibrational absorption of adenine, guanine, saturated lipids and amides in proteins [23]. At the same time, as shown in Figure 10b, the peak intensity at 733 cm^−1^ remains stable, and the relative standard deviation (RSD) is calculated to be 6.1%, indicating the good homogeneity or repeatability of the as-prepared Ag@T-A@SiO_2_@Au-20 substrates.

So far, it has been confirmed that the as-prepared electrospun nanofibrous membrane SERS substrate has excellent sensitivity, good stability, and superior repeatability. Furthermore, the relationship between the SERS peak intensity and the concentration of the bacterial suspension was established in order to realize the quantitative analysis of bacteria. Figure 10c shows the SERS spectra of *S. aureus* with different concentrations (from 10^3^ to 10^8^ cfu/mL). As seen from Figure 10c, the spectra of different concentrations of *S. aureus* adsorbed on the Ag@T-A@SiO_2_@Au-20 nanofibrous membranes show significant enhanced Raman signals. Taking the characteristic Raman peak at 733 cm^−1^ as an example, it can be seen that the peak intensity decreased gradually with the decrease in bacterial concentration. However, this SERS peak can be clearly distinguished even if the concentration is as low as 10^3^ cfu/mL. More importantly, Figure 10d shows the relationship plot between the peak intensity and the logarithm of *S. aureus* concentration. It can be seen from Figure 10d that there is a good linear correlation between the peak intensity at 733 cm^−1^ and the logarithm of bacterial concentration, and the correlation coefficient is calculated to be 0.9461. Compared with the related literature [44], the result of this work is superior. Based on the above SERS analysis results for bacteria, it is demonstrated that the as-prepared Ag@T-A@SiO_2_@Au-20 nanofibrous membrane substrate can directly obtain the characteristic Raman spectra of bacteria without a complicated ligand binding process, and possess ultra-high detection sensitivity and excellent uniformity. It is worth emphasizing that in addition to qualitative identification, this substrate can also perform quantitative SERS detection of biological macromolecules, making it more practical for application.

## 4. Conclusions

In the present study, we have developed Ag@T-A@SiO_2_@Au nanofibrous membranes SERS substrates with synergistic Raman enhancement of bimetal via a combined process of electrospinning and surface chemical modification. The structure and morphology of the as-prepared nanofibers were characterized by techniques such as TEM, SEM, AFM, XRD, EDS, XPS and TGA. It is found that Au nanoparticles with an average particle size of 8 nm are uniformly incorporated into SiO_2_ nanofibers, while Ag nanoparticles with diameters of 33 nm are densely and uniformly deposited of the surface of the nanofibers. The as-prepared flexible nanofibrous membranes by the synergistic Raman enhancement of Au/Ag bimetals not only form a lot of hot spots, but also can firmly capture a variety of analyte molecules, and thus this is an ideal substrate for SERS detection. Using small probe molecules and pesticide as target analytes, SERS effects of Ag@T-A@SiO_2_@Au composite nanofibers are investigated, and this substrate allows the detection of 4-MPh, 4-MBA and thiram, at low concentrations of 10^−11^ and 10^−8^ mol/L, respectively (i.e., showing an ultra-high SERS sensitivity). The EF is calculated to be 10^8^ for small probe molecules. Furthermore, the as-prepared nanofibers exhibit excellent SERS signal stability in the durability test, the peak intensity remains detectable for five washes or lasting for 60 days. More importantly, this flexible and free-standing Ag@T-A@SiO_2_@Au nanofibrous SERS substrate can directly identify *S. aureus* without previous bacteria−aptamer conjugation. The detection limit is 10^3^ cfu/mL, and a fine linear relationship of peak intensity and bacterial concentration in the range of 10^3^ to 10^8^ cfu/mL is obtained, making it suitable for quantitative analysis. Meanwhile, the substrate demonstrated outstanding repeatability for *S. aureus* detection because of its homogeneous structure. Summarily, it is believed that the versatile Ag@T-A@SiO_2_@Au electrospun nanofibrous SERS substrate developed herein can be expected to have great practical application potential in the trace detection of chemical and biological molecules.

## Supplementary Materials

The following are available online at https://www.mdpi.com/2073-4360/12/12/3008/s1, Figure S1: Diameters distribution histograms for nanofibers, Figure S2: Particles size distribution histograms for nanoparticles, Figure S3: AFM images of nanofibrous membranes, Figure S4: SEM images of bacteria attached on the membranes, Table S1. EF of the as-prepared Ag@T-A@SiO_2_ nanofibrous substrate, Table S2. EF of the reported electrospun SERS substrates.
