# Supplementary material for: Loading of Au/Ag Bimetallic Nanoparticles within and Outside of the Flexible SiO2 Electrospun Nanofibers as Highly Sensitive, Stable, Repeatable Substrates for Versatile and Trace SERS Detection
Source: Polymers (Basel). 2020 Dec 16;12(12):3008. doi: 10.3390/polym12123008 (PMC7766957; doi:10.3390/polym12123008)
Supplement: Supplementary file 1 [file polymers-12-03008-s001.pdf]

Supplementary material

Loading of Au/Ag bimetallic nanoparticles within and outside of the flexible SiO_2 electrospun nanofibers as highly sensitive, stable, repeatable substrates for versatile and trace SERS detection

Menghui Wan, Haodong Zhao, Lichao Peng*, Xuanyan Zou, Yanbao Zhao and Lei Sun*

Engineering Research Center for Nanomaterials, Henan University, Kaifeng 475004, China; wanmenghui950825@163.com (M.W.); zhd880880@163.com (H.Z.); zouxueyan@henu.edu.cn (X.Z.); zhaoyb902@henu.eud.cn (Y.Z.)

* Correspondence: plc@henu.edu.cn (L.P.); sunlei@henu.edu.cn (L.S.)

Received: 20 November 2020; Accepted: 14 December 2020; Published: XX December 2020

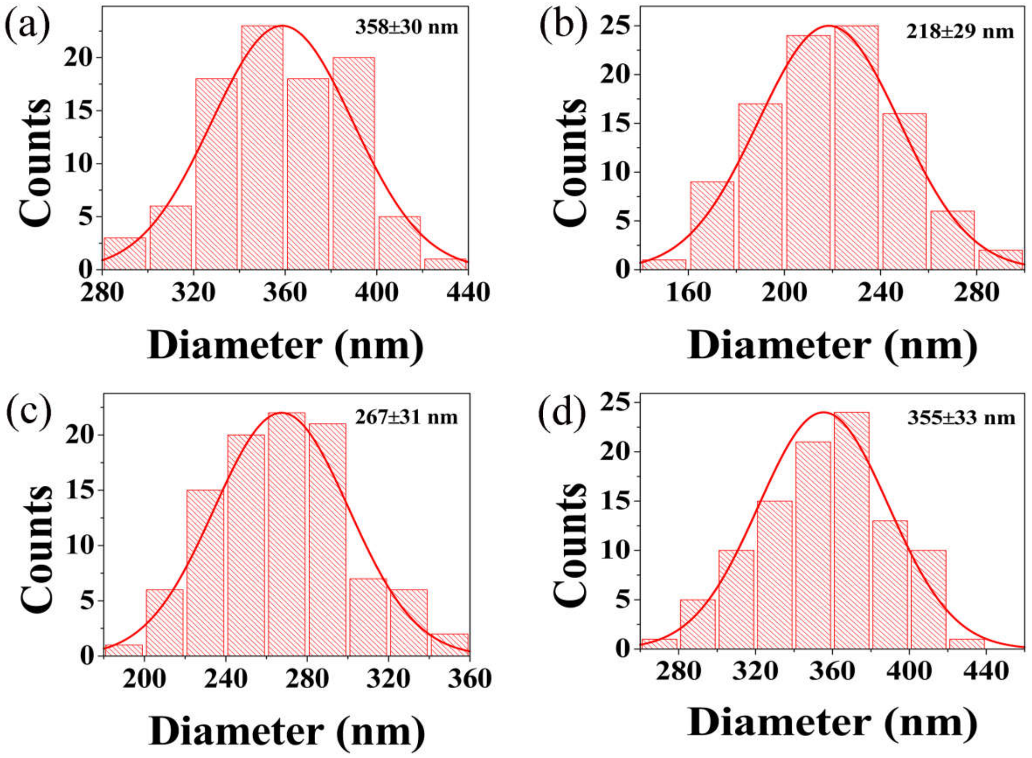

Figure S1. Diameters distribution histograms for nanofibers of SiO_2@Au-20 precursor (a), SiO_2@Au-20 after calcination (b), T-A@SiO_2@Au-20 (c), and Ag@T-A@SiO_2@Au-20 (d).

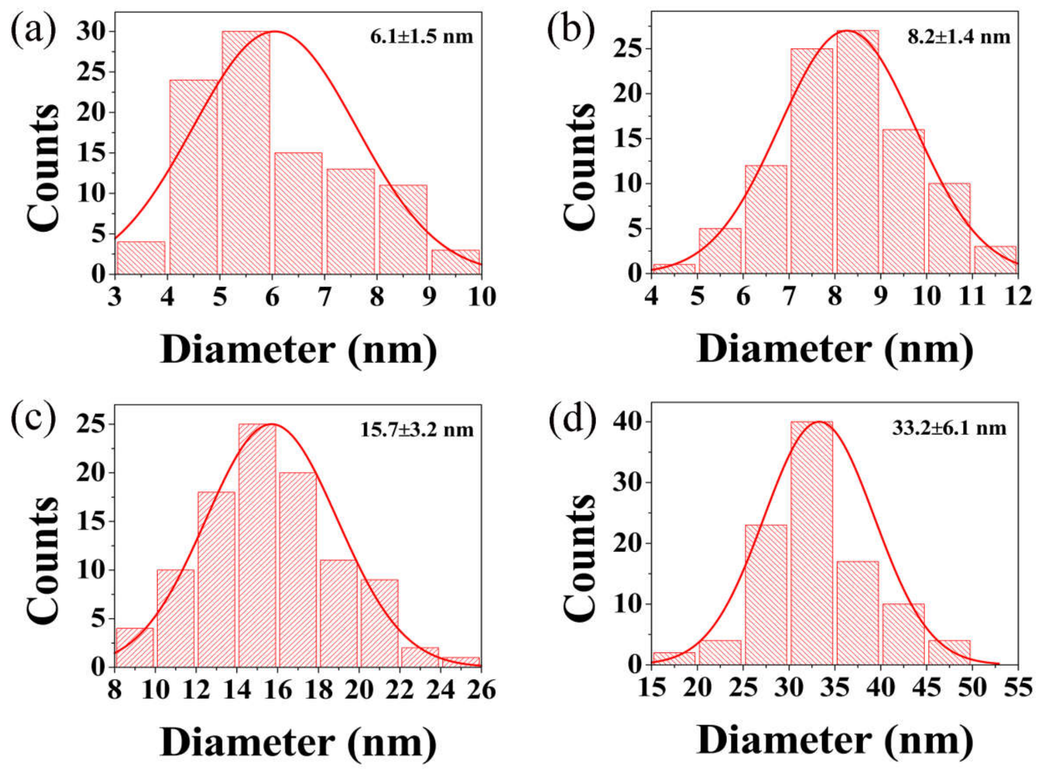

Figure S2. Particles size distribution histograms for Au nanoparticles incorporated into the SiO_2@Au-10 (a), SiO_2@Au-20 (b), SiO_2@Au-30 (c) nanofibers, and histograms for Ag nanoparticles decorated on the surfaces of Ag@T-A@SiO_2@Au-20 nanofibers (d).

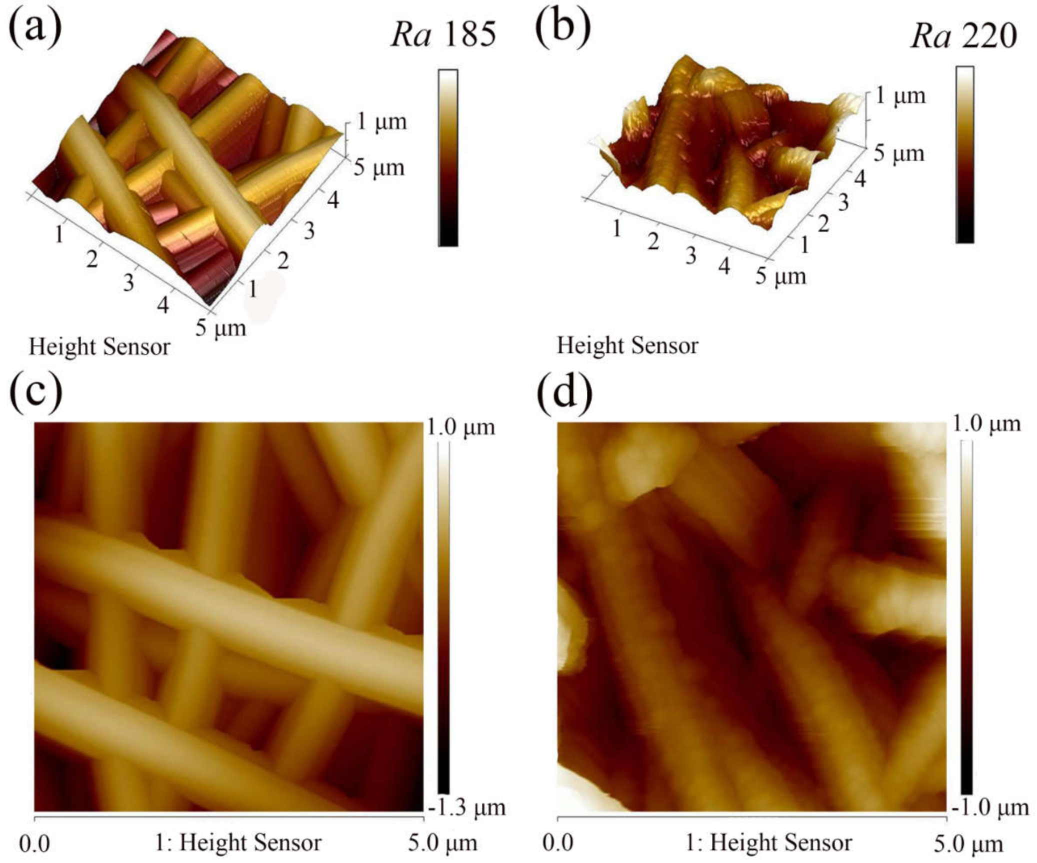

Figure S3. Three-dimensional (3D) and two-dimensional (2D) AFM images of SiO_2@Au-20 (a, c) and Ag@T-A@SiO_2@Au-20 (b, d) electrospun nanofibrous membranes.

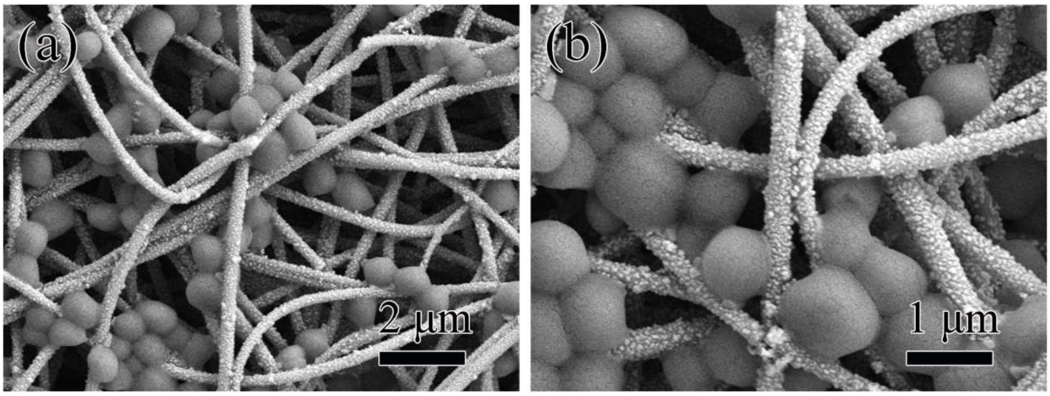

Figure S4. SEM images of different magnifications for *S aureus* attached on the Ag@T-A@SiO_2@Au-20 nanofibrous membranes (a, b).

Table S1. The enhancement factor (EF) of probe molecule on Ag@T-A@SiO_2-20 nanofibrous substrate

	**4-MPh**	**4-MBA**
**Raman Shift (cm^{-1})**	1073	1587
I_{NR}	309	1453
I_{SERS}	1680	3396
EF	5.4×10^8	2.3×10^8

Table S2. The enhancement factor (EF) of the reported electrospun SERS substrates

**Electrospun SERS Substrates**	**Analytes**	**EF**	**References**
TiO_2/Ag	4-mercaptobenzoic acid	5.62×10^6	[3]
ASFPAN-Ag NPs	4-mercaptobenzoic acid	4.8×10^5	[33]
AgNPs/Agar/PAN	p-aminothiophenol	3.1×10^5	[32]
